# Noise-Aided Logic in an Electronic Analog of Synthetic Genetic Networks

**DOI:** 10.1371/journal.pone.0076032

**Published:** 2013-10-04

**Authors:** Edward H. Hellen, Syamal K. Dana, Jürgen Kurths, Eli Kehler, Sudeshna Sinha

**Affiliations:** 1 Department of Physics and Astronomy, University of North Carolina Greensboro, Greensboro, North Carolina, United States of America; 2 Council of Scientific and Industrial Research-Indian Institute of Chemical Biology, Kolkata, India; 3 Potsdam Institute for Climate Impact Research, Potsdam, Germany; 4 Indian Institute of Science Education and Research Mohali, SAS Nagar, Mohali, Punjab, India; University of Adelaide, Australia

## Abstract

We report the experimental verification of noise-enhanced logic behaviour in an electronic analog of a synthetic genetic network, composed of two repressors and two constitutive promoters. We observe good agreement between circuit measurements and numerical prediction, with the circuit allowing for robust logic operations in an optimal window of noise. Namely, the input-output characteristics of a logic gate is reproduced faithfully under moderate noise, which is a manifestation of the phenomenon known as *Logical Stochastic Resonance*. The two dynamical variables in the system yield complementary logic behaviour simultaneously. The system is easily morphed from AND/NAND to OR/NOR logic.

## Introduction

Realization of logic functions in different physical systems is one of the key questions that commands widespread research interest in science and engineering. Universal general-purpose computing devices can be constructed entirely from NOR/NAND logic gates [1], [2]. It is particularly interesting to investigate if systems of biological relevance can also yield logic outputs consistent with the truth tables of different logic functions (see Table 1). Biological systems are capable of stochastic resonance[3]–[6], a process in which a small signal is amplified due to the presence of an appropriate level of noise, leading to the possibility of a biological system performing robust noise-aided logic operations in response to weak input signals.

**Table 1:** Logic Table.

Input Set ( 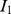 , 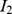 )	OR	AND	NOR	NAND
(0,0)	0	0	1	1
(0,1)/(1,0)	1	0	0	1
(1,1)	1	1	0	0

Relationship between the two inputs and the output of the fundamental OR, AND, NOR and NAND logic operations. Note that the four distinct possible input sets 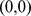, 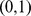, 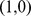 and 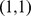 reduce to three conditions as 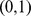 and 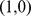 are symmetric. Note that *any* logical circuit can be constructed by combining the NOR (or the NAND) gates [1], [2].

A new idea in this direction uses the interplay between noise and nonlinearity constructively to enhance the robustness of logic operations. Namely, in an optimal window of noise, the input-output characteristics of a logic gate is reproduced faithfully. This phenomenon is termed *Logical Stochastic Resonance* (LSR) [7]–[12]. Specifically, in LSR we consider the state of a nonlinear system when driven by input signals, consisting of two randomly streaming square waves. It was observed that the response of such a system shows a remarkable feature: in an optimal band of noise, the output of the system, determined by its state, is a logical combination of the two input signals in accordance with the truth tables of fundamental logic operations.

An important motivation for further studying LSR stems from an issue that is receiving widespread attention currently. The number of transistors in an integrated circuit has approximately doubled every year in accordance with Moore’s law. The rapid shrinking of computing platforms with smaller power supplies has brought with it problems of smaller noise margins and higher error rates. Namely, as computational devices and platforms continue to shrink in size, we encounter fundamental noise that cannot be suppressed or eliminated. Hence an understanding of the cooperative behavior between a device noise-floor and its nonlinearity plays an increasingly crucial role in paving the way for smart computing devices. In this direction, LSR indicates a way to turn potentially performance degrading noise to assist the desired operation. Further, it is of far reaching interest to obtain analogous behaviour, not merely in human engineered physical systems, but also in systems of chemical and biological relevance, in order to explore the information processing capacity of naturally occurring systems where noise is ubiquitous.

Since the idea of LSR was first introduced [7], several systems implementing and displaying LSR have been found. To begin with, the basic electronic realizations of simple bistable potentials were reported [7], [8]. Subsequently, noise-aided reprogrammable logic gates have been implemented with noisy nanomechanical oscillators [12], chemical systems [12] and optical systems [13], [14].

Most recently, in the context of biological systems, theoretical ideas have been proposed [15]–[18] on the implementation of LSR in a synthetic genetic network [19]. Now, in this work, we will provide *experimental realizations* of these ideas in an electronic analog of a noisy synthetic gene network. Specifically then, we will investigate the possibility of obtaining reliable logic outputs, and *explicitly demonstrate the pivotal role of noise in the optimization of the logic performance in this circuit*. Further, we will show that the system is easily changed from AND/NAND logic to OR/NOR demonstrating potential for re-programmability [15], [16]. Our results will thus provide verification and further understanding of noise aided logic in systems that are of considerable importance in biology.

Since understanding the intracellular processes in a network of interacting biomolecules is difficult, an alternative approach has been started recently [20], to design artificial genetic networks to derive desired functional behaviors. One important early design is a clock using three genes inhibiting each other in a cyclic order [21]. Taking into account the standard chemical kinetics for expression, degradation and inhibition, a dynamical system model was proposed where the repressor-protein concentrations and mRNA concentrations were expressed as dynamical variables. Another design is a synthetic genetic toggle-switch network [22] whose potential for noise-aided logic operation is investigated here.

The significance of using both numerical simulation and electronic circuits to model a potential synthetic genetic network is two-fold. Firstly, the numerical and circuit methods provide *different* imperfect models of a potential biological system. Agreement between these two models indicates robustness in the system and therefore greater likelihood that the same behavior could be realized in the proposed biological system. The biological system is generally much more difficult to construct, and therefore investigating proposed networks in simpler systems is prudent. Secondly, modeling with stochastic differential equations is nontrivial compared to ordinary differential equations, so that the addition of experimental measurements from a physical system such as an analog circuit provides valuable verification. Thus the circuit is an additional tool for investigating potentially interesting biological networks in the presence of noise.

Here we use two repressors and constitutive promoters as our model system for implementing logic functions. We begin with a brief description of LSR, then we describe the synthetic gene network model, and define what constitutes logic inputs and logic outputs in this system. We then go on to present the electronic analog of the system followed by a comparison of numerical simulation and experimental measurement.

## Methods

We begin with a short description of the general principle of LSR. Consider a general nonlinear dynamic system, given by
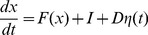
(1)where 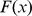 is a generic nonlinear function which has or nearly has two distinct stable energy wells. 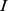 is a low amplitude input signal and 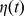 is an additive zero-mean Gaussian noise with unit variance with 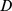 being the noise strength.

We achieve a logical input-output correspondence with such a system by encoding N inputs in N square waves. Specifically, for two logic inputs, we drive the system with a low amplitude signal 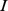, taken to be the sum of two pulse trains: 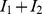, where 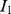 and 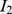 encode the two logic inputs. Now the logic inputs can be either 0 or 1, giving rise to 4 distinct logic input sets 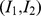: (0,0), (0,1), (1,0) and (1,1). Since the input sets (0,1) and (1,0) give rise to the same 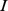, the input signal generated by adding two independent input signals is a 3-level aperiodic waveform.

The output of the system is determined by its state. For instance, for a bistable system with wells at 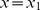 and 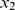, the output can be considered a logical 1 if it is in the well at 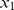, and logical 0 if it is in 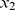. If we consider the opposite assignment, namely logical 1 if the state is in well 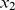 and logical 0 if the state is in well 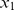, we obtain a complementary logic operation. Specifically we can have an output determination threshold 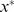, located near the barrier between the wells, and the logical outputs are then simply given by the state being greater than or less than 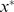. It is possible that the input 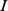 induces the appearance of the second energy well if it was not already there.

The central result of LSR is as follows: for a given set of inputs 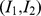, a logical output, in accordance with the truth tables of the basic logic operations, is consistently obtained only in an optimal window of noise. Namely, under very small or very large noise the system does not yield reliable logic outputs, while in a band of moderate noise it produces the desired output.

### Synthetic Genetic Network Model and Logic Operation

We consider the previously used variation [15] of the genetic toggle switch model comprised of two genes inhibiting each other [22]. The concentrations of the two expressed proteins are 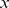 and 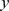, and their rates of change are:
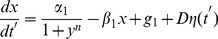
(2)
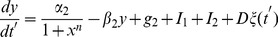
(3)where 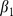, 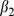 are the rates of decay of each expressed protein and 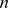 is the Hill coefficient. The 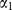, 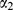 describe the maximum expression rates in absence of inhibitor and they are used here as tunable parameters. In the original model 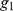 and 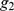 represent the basal synthesis rates of the promoters [23], however we use them as constant bias. The additive noise has strength 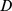 and 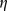 and 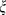 are chosen from unit variance zero mean Gaussian distributions. Such an additive noise source alters the background repressor production and represents the inherent stochasticity of biochemical processes such as transcription and translation, and the fluctuations in the concentration of a regulatory protein. 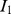 and 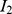 are two *low amplitude* inputs providing independent parallel production pathways of repressor *y*. The 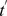 indicates dimensionless time.

**Figure 1:**
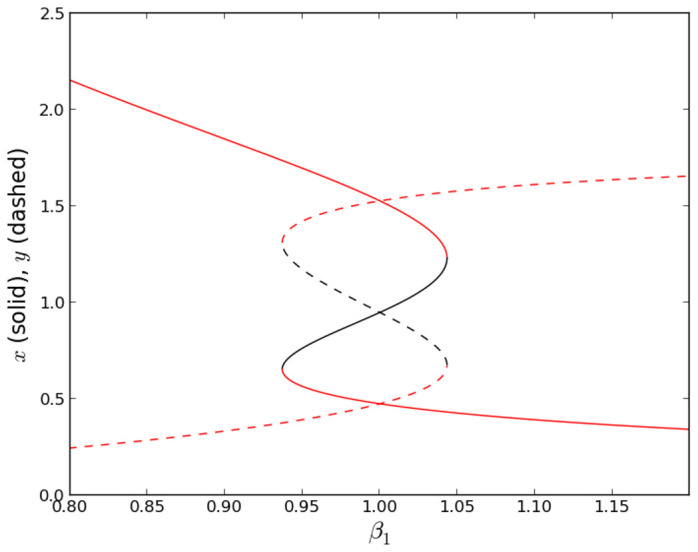
Bifurcation diagram for state-variables 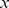 (solid) and 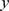 (dashed). 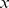
 and 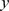 are complementary outputs, when one is high the other is low. Red (black) indicate stable (unstable) fixed points. System is bistable for 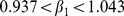. For 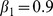, 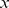 is high and 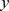 is low. Calculated for Eqs. (2)–(3) with 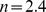, 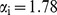, 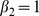, 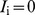, 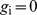, and 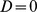.

The system above may have two stable configurations in the 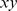-plane: one state has a high value of 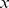 (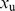) and a low value of 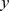 (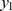); the other state has a low value of 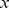 (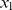) and a high value of 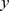 (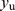). That is, the two dimensional potential underlying this system has two wells, 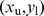 and 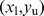, in the 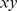-plane. Varying the parameters changes the depth and position of these wells, and also determines whether there are one or two wells. For example, Fig. 1 shows that for the case 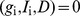 the system in Eqs. (2)–(3) is bistable and therefore has two stable wells only when 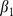 is close to 1.

#### Encoding inputs

Here the low amplitude input signal is 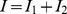, with 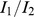 equal to 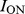 (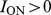) if the logic input is 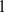, and 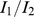 being 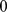 if the logic input is 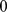. So we have:

(i) 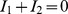 corresponds to logic input set 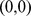.

(ii) 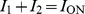 corresponds to logic input sets 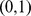/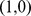.

(iii) 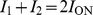 corresponds to logic input set 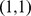.

#### Output

The outputs of the system are determined by the level of the dynamical variables 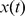 and 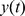. For instance the output can be considered a logical 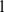 if the state is at the high level, and logical 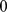 if it is at the lower level. That is:

(i) If 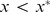, then Logic Output is 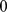.

(ii) If 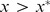, then Logic Output is 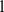.

Here 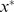 is the *output determination threshold* that lies between the two states, e.g., at the position of the barrier between the wells. The results presented here are not sensitive to the specific value of 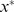.

Specifically, in this work, we consider the logic output to be 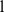 when the state is close to the upper well, and 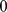 when the state is close to the lower well, for both 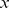 and 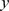 variables. So when the system switches wells, the output is “flipped” or “toggled”.

The model in Eqs. (2)–(3) is based on the synthetic genetic toggle switch previously expressed in *E. coli* [22]. Parameter values used in [22] correspond here to 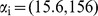, 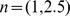, and 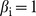 in Eqs. (2)–(3). By comparison, here we use 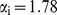, 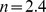, 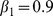, and 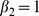. The bifurcation diagram in Fig. 1 indicates that these parameter values, along with 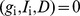, result in a system with a single stable well at 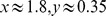. A non-zero input 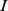 can then “shift” the bifurcation diagram of Fig. 1 so that there is a stable state with low-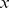, high-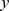 for 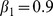.

### Circuit Realization

**Figure 2:**
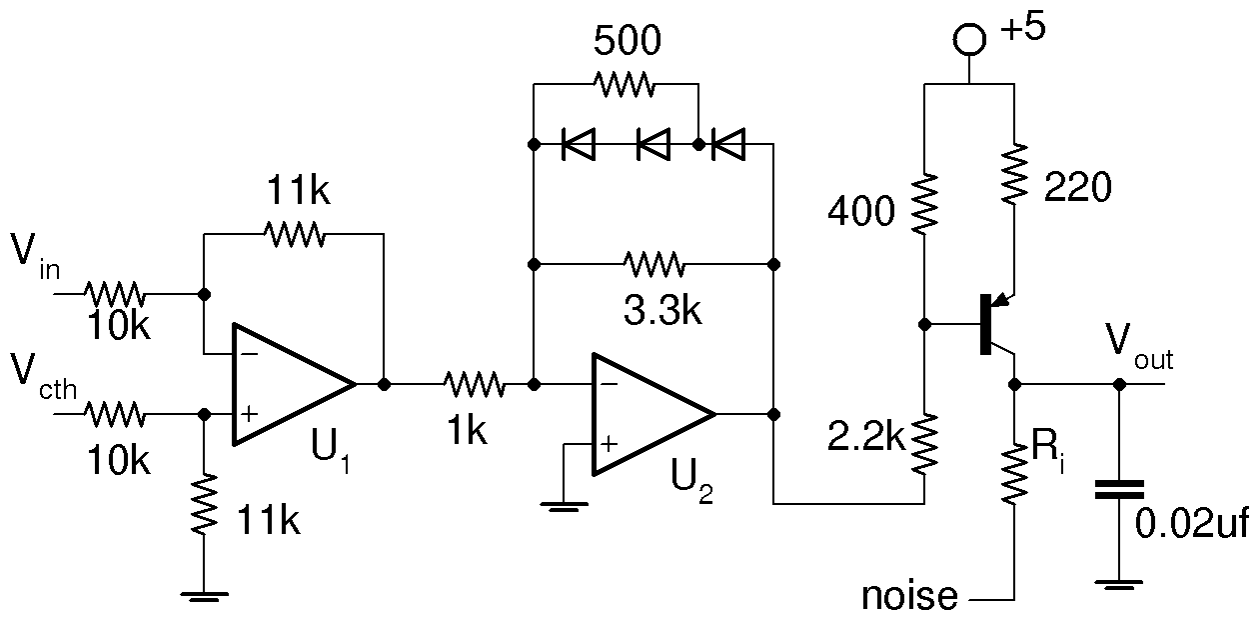
Circuit for single gene. Inhibitory input at 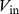. Expressed protein concentration is represented by 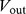. 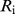 = 470 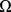 for gene-*y*, 520 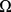 for gene-*x*. Dual op-amp is LF412 supplied by +/−5 V. The *pnp* transistor is 2N3906. The input noise has a mean of 0 V (gnd) and controllable standard deviation.

**Figure 3:**
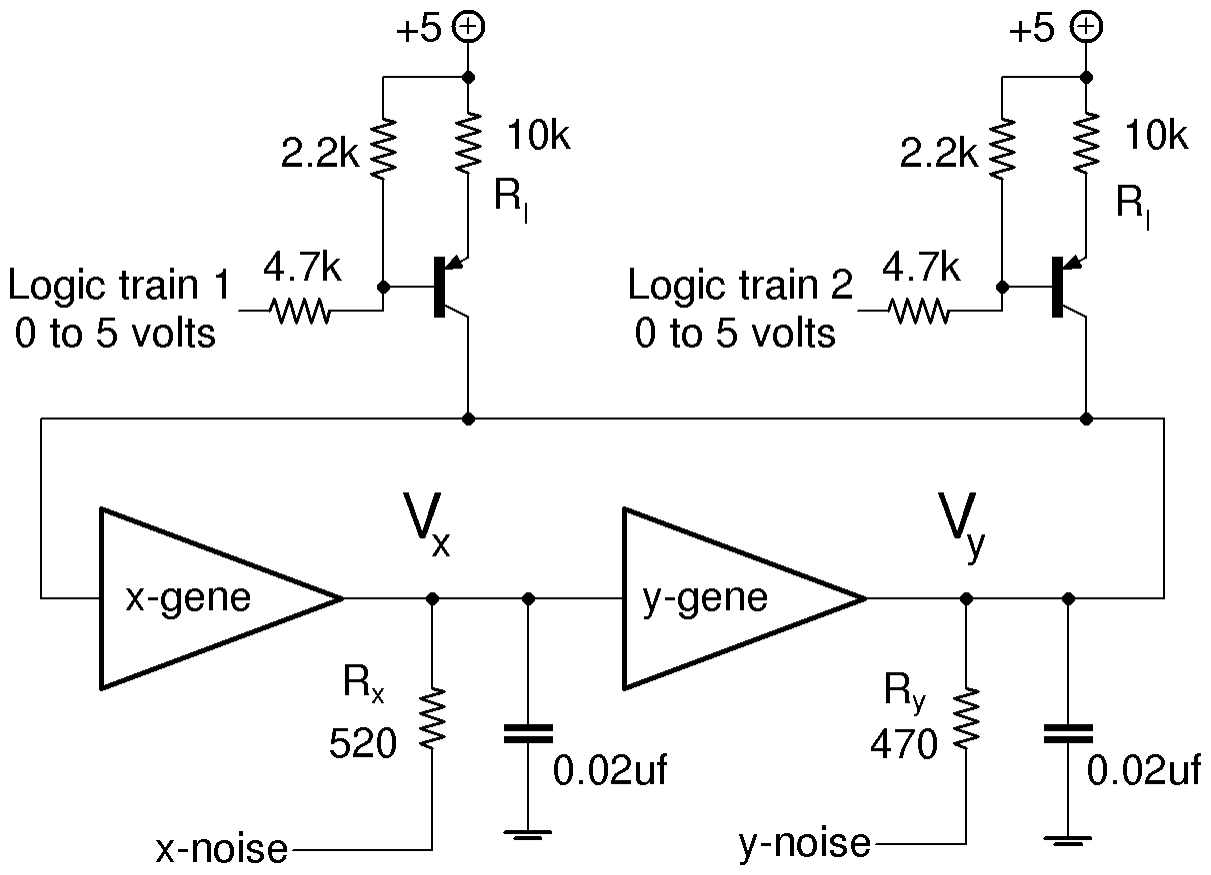
Circuit for synthetic genetic network. Encoding inputs are 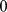 to 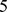 V pulse trains creating transistor currents of 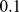 and 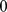 mA, respectively, for 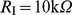. The *x* and *y* gene circuits are shown in Fig. 2. Each noise input is connected to its own noise circuit shown in Fig. 4.

The circuit of a single inhibitory gene [24], [25] is shown in Fig. 2. The transistor current represents the rate of gene expression and the voltage 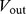 represents the concentration of expressed protein. 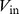 represents the concentration of repressor, and the 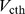 adjusts the affinity of the repressor binding to the gene’s DNA. The Hill function inhibition in Eqs. (2)–(3) is accounted for by the dependence of the transistor current on repressor concentration voltage 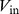. The synthetic genetic network shown in Fig. 3 is comprised of two individual gene circuits connected in a loop, each inhibiting the other. For the model in Eqs. (2)–(3), the encoding inputs 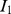 and 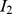 add to production of 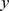 which is accounted for in Fig. 3 by the two logic-driven transistors sourcing current to 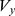. Initially parameters 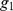 and 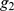 are taken to be zero.

**Figure 4:**
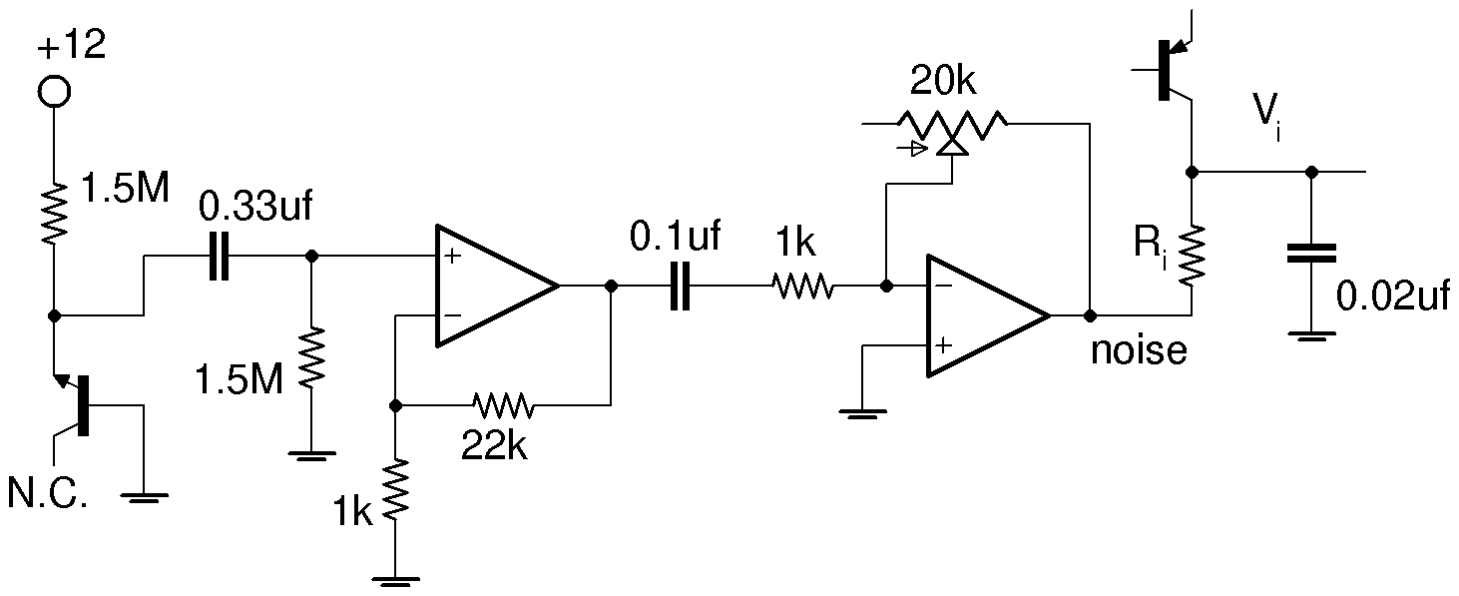
Noise circuit and its connection to resistor of gene circuit. Source of noise is the reverse-biased base-emitter junction of the 2N3904 *npn* transistor on left. OPA2228 dual op-amps supplied from +/−12 V regulators. OPA2228 has gain-bandwidth product of 33 MHz.

The circuit equations are obtained by applying Kirchoff’s laws to 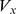 and 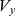, the voltages across the capacitors in Fig. 3 [24], [25]. Multiplying both equations by 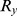 results in equations for 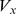 and 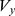;
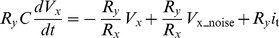
(4)
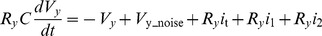
(5)where 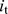 are the Fig. 2 transistor currents for each gene, and 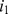 and 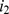 are currents from the logic train transistors in Fig. 3. A noise generation circuit shown in Fig. 4 based on breakdown of a reverse biased base-emitter junction produces noise 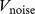 with zero mean and variable amplitude. We use a well regulated supply for the noise circuit to avoid adding AC signals from the building’s electrical system into the noise. Two of these noise circuits are used to supply noisy voltages to each individual gene at locations indicated in Fig. 3.

The connections between model parameters (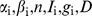) and circuit parameters are presented in this section using relevant numerical values, with derivations of these connections given in the next section. Readers may go directly to *Results and Discussion* without loss of continuity. The connections are found by relating circuit Eqs. (4)–(5) to the model Eqs. (2)–(3) and by adjusting the dependence of the transistor current 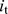 on 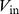 in Fig. 2 to match the Hill function inhibition. The dimensionless state-variables (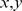) in Eqs. (2)–(3) are related to voltages 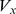 and 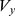 by:
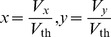
where 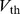 corresponds to the repressor’s half-maximal inhibition binding constant 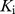. The maximal expression rate is
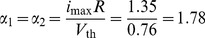
(6)where the voltage 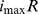 is 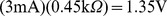 and 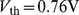. Protein decay rates are 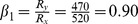, and 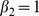. The Hill coefficient 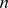 comes from
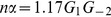
(7)where 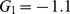 and 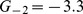 are closed loop gains of U1 and U2 in Fig. 2 resulting in 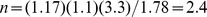. The characteristic time is 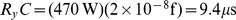, so the dimensionless time is 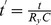.

The high value 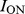 of the encoding signals 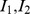 is given by
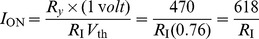
(8)where 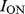 is changed by varying 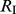 in Fig. 3, i.e. 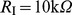 gives 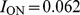. A non-zero value of 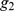 in Eq. 3 is achieved in the circuit by including a third current-sourcing transistor in Fig. 3 in the same way as the two encoding signal transistors, but with the emitter resistor labelled 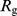 and the input grounded so that the transistor provides a constant current 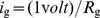. 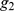 is changed by varying 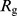 in the same way 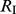 controls 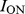. The non-zero 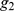 adds a term 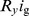 to Eq. 5, where


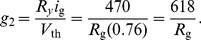
(9)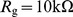 gives 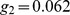.

The 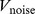 terms in Eqs. (4)–(5) approximate white noise voltages. Each 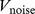 is characterized by its measured 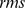 value 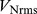 and bandwidth. 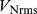 is controlled by changing the gain via the potentiometer in Fig. 4. Noise strength 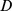 in Eqs. (2)–(3) is given by
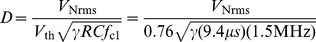
(10)where 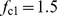 MHz is the cut-off frequency for the amplifier in Fig. 4 and 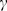 decreases from 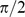 at low gain to 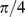 at high gain (when the potentiometer is set to 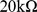 in Fig. 4).

### Circuit Analysis and Simulation

Here we describe the circuit analysis and derive the connections between the model parameters used in Eqs. (2)–(3) and the circuit parameters. Further details are given in Refs. [24], [25].

The single gene circuit in Fig. 2 is designed to reproduce the Hill function inhibition in Eqs. (2)–(3). The op-amp U1 is configured as a subtraction amplifier with gain 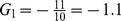. Replication of the Hill function behavior is achieved by allowing saturation of the output of the op-amp U2 and by having different unsaturated gains 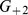 and 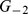 for 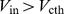 and 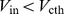, respectively, due to the diodes in the feedback for U2. 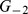 is the gain of U2 when its output goes negative, in which case the diodes are not conducting, and therefore 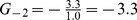. 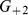 is a diminishing gain when the output of U2 becomes increasingly positive causing the diodes to go into conduction. An increasing repressor concentration corresponds to 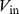 surpassing 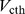 which causes the unsaturated output at U2 to change from a negative voltage of 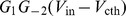 to a positive voltage 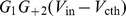. The increasing voltage at the output of U2 turns the transistor off (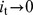) which corresponds to complete inhibition of protein expression. Maximal protein expression 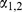 in Eqs. (2)–(3) corresponds to the maximum value of 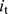, designated 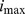. 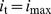 occurs when 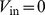 (no repressor) because the output of U2 saturates at 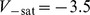 V (for the LF412 op-amp supplied by 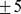 V), resulting in a 0.65 V drop across the 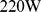 and therefore 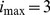 mA.

Circuit parameters for 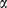 and 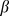 are found by using 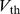 to convert Eqs. (4)–(5) to a dimensionless form for comparison to Eqs. (2)–(3). The 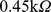 used for 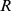 in Eq. (6) comes from the 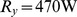 being nearly in parallel with the resistance 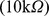 at the input to the subtraction amplifier U1 for gene-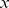. The relation for Hill coefficient 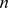 is found by adjusting the dependence of 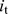 on 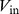 to match the slope of the Hill function 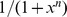 at 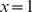 resulting in the relation [25]:
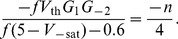
(11)

In Fig. 2 the voltage divider fraction 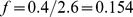 and 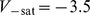 V. Using Eq. (6) in Eq. (11) yields Eq. (7).

Parameter 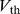
’s correspondence to binding affinity of repressor to DNA is seen by noting that Eqs. (2)–(3) are dimensionless, meaning that in the process of going from chemical kinetic equations to Eqs. (2)–(3) the maximal expression rate 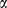 has been scaled by the repressor’s inhibition binding constant 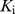, and by a mRNA degradation rate [21], [25]. From Eq. (6) it follows that 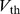 is proportional to 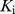 since 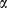 is inversely proportional to 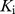 due to the scaling. To find the relation between 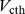 in Fig. 2 and 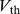 we note that the Hill function equals 0.5 when 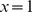. Therefore 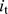 must be half its maximum value when 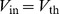 which gives [25]
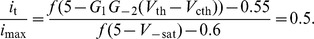


Solving gives 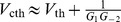. Using 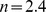 and 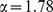 in Eqs. (6)–(7) gives: 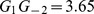, satisfied by 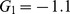 and 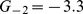; 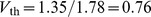 V; and 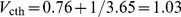 V.

Comparing Eqs. (3) and (5) shows that the encoding signals 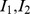 are related to the transistor currents 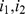 by:
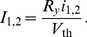
(12)

The encoding currents 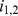 take on two possible values depending on whether their logic train input in Fig. 3 is high or low. When the input is high (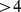
V) the transistor is off so the current is zero. When the input is zero, the voltage divider consisting of the 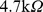 and 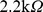 produces one volt across the 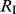 connected to the emitter of the *pnp* transistor creating current 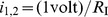. Equation (12) then gives Eq. (8) for 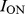. Results of an analysis for a non-zero value of parameter 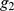 are the same as for encoding signals 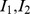 and currents 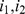 because 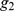
’s sourcing transistor is set up in the same way as the transistors in Fig. 3 for the encoding signals. Thus the non-zero value of 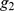 is Eq. (9).

Here we show how to use simulations to predict the circuit results. In the process we find Eq. (10), the connection between circuit parameters and the noise strength 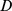 in Eqs. (2)–(3). A standard Euler-Maruyama simulation of Eq. (2) is
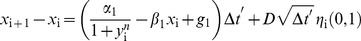
(13)where 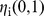 is a unit variance zero mean normal random distribution. The noise circuit in Fig. 4 produces a measurable *rms* voltage 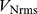 consisting of contributions from all the frequency components present in the noise. The variance of the noise is the integral of its spectral density function 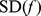 over frequency, and is obtained from a measurement of 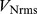;


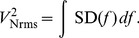
(14)Idealized white noise assumed in Eqs. (2)–(3) has a 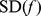 which is uniform over an infinite bandwidth. However for the real noise from the 2-stage noise amplifier circuit in Fig. 4 each op-amp’s gain-bandwidth product produces a high frequency cut-off, 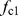 and 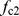. The resulting 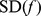 has the form
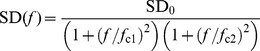
(15)where 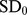 is a constant related to the strength of the noise. The OPA228 op-amp has a gain-bandwidth product 33 MHz, therefore the first stage in Fig. 4 with fixed gain 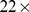 has cut-off, 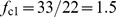 MHz. The second stage’s cut-off 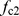 varies from 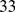 to 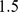 MHz depending on the gain setting determined by the potentiometer in the feedback of the second stage op-amp.

The integration in Eqs. (14)–(15) gives
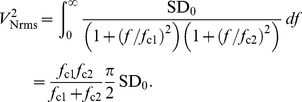
(16)

There are two limiting cases for the integral: for small gain 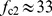 MHz 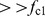 giving 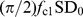; and for large gain (potentiometer 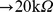 in Fig. 4) 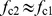 giving 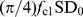. Thus the integral in Eq. (16) is 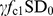 where 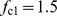 MHz and 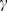 varies from 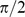 for small noise to 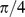 for large noise. For frequencies within the noise bandwidth (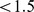 MHz) the amplitude spectral density ASD (units Volt Hz-1/2) has a constant value given by
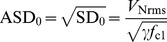
(17)


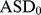
 is a good approximation of the white noise strength provided that the Fig. 3 genetic circuit’s characteristic response rate 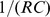 is much less than the noise bandwidth, meaning that 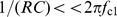. This condition is ensured since the 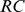 used here is 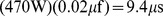, and the bandwidth of the noise is 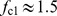 MHz.

**Figure 5:**
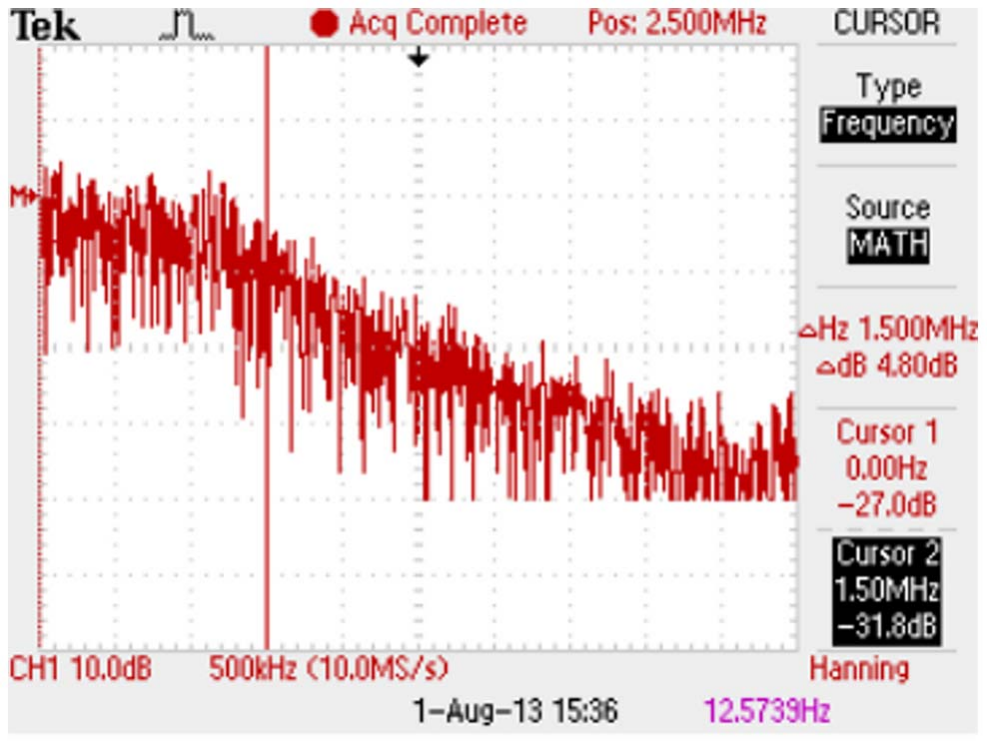
Measured frequency spectrum of noise. For 2-stage noise amplifier shown in Fig. 4 with second stage gain 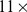. Horizontal scale is 500 kHz/Div, so cursor indicates 1.5 MHz as location of cut-off frequency. From FFT function on Tektronix TDS 2024B oscilloscope.

Figure 5 shows the measured frequency content from the noise circuit when the potentiometer at the second stage is set for gain 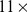 producing 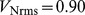 V and 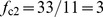 MHz. In this case 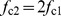 and 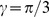. Figure 5 shows that the frequency content is relatively flat out to the cut-off near 1.5 MHz and therefore is a good approximation to white noise for the genetic network circuit. 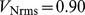 V is on the high end of the noise amplitudes used here.

The circuit Eqs. (4)–(5) which need to be simulated are of the form
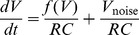
(18)where 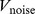 approximates a white noise voltage. 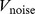 is characterized by its measured 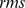 value 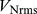 and bandwidth, and Eq. (17) gives the noise’s amplitude spectral density. The Euler-Maruyama simulation for Eq. (18) is


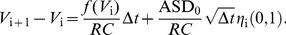
(19)Using dimensionless time 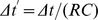 and the measured noise amplitude 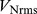 gives
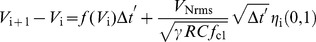
(20)

Normalizing by the voltage scale 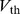 puts Eq. (20) in the form of Eq. (13) and gives the connection between circuit parameters and dimensionless noise amplitude 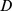 shown in Eq. (10). For example, using 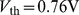, 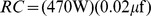, 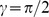, and 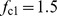 MHz gives 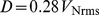.

## Results and Discussion

**Figure 6:**
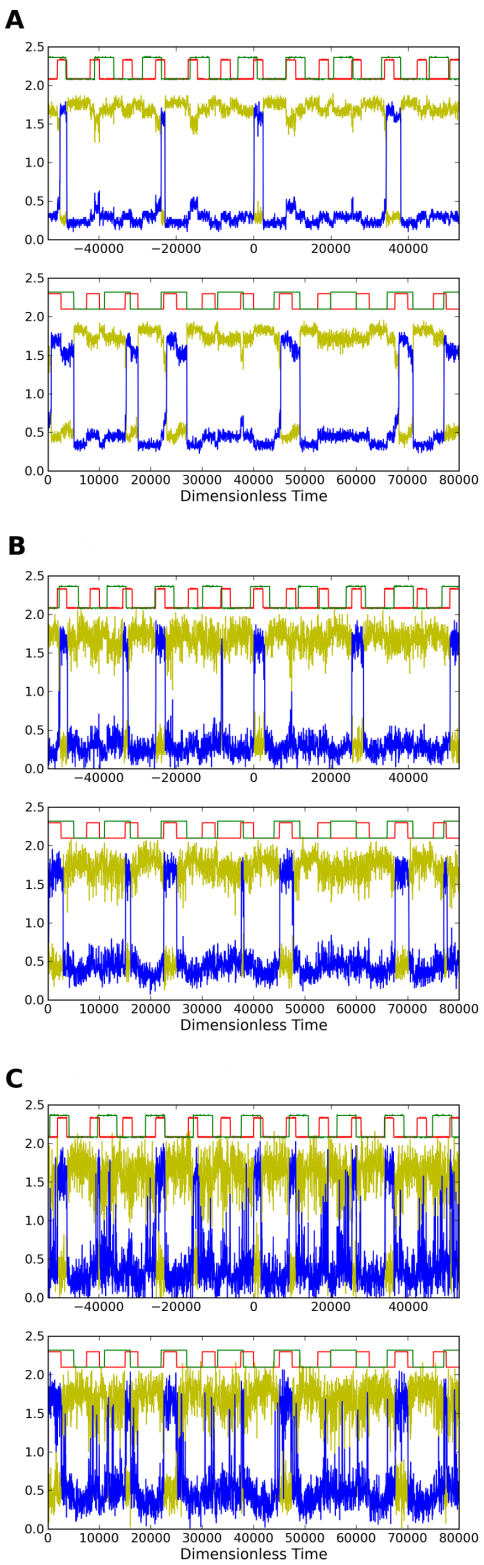
Time series for circuit measurements (upper graph) and simulations (lower) for different noise strengths showing AND/NAND LSR. Circuit shown in Fig. 3. Simulations are of Eqs. (2)–(3). Upper red and green traces indicate logic states of the encoding inputs 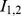 and lower traces show the complementary outputs 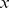
(yellow) and 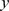
(blue). Panel (b) has noise level within the optimal range for displaying AND/NAND characteristics. Noise strengths in simulation and in circuit are: (a) 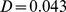 and 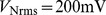, (b) 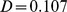 and 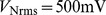, (c) 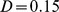 and 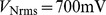. Voltages and times for the circuit measurements have been converted to dimensionless quantities as described in the text.

Figure 6 shows simulations and circuit measurements for three values of noise using parameters 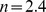, 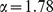, 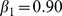, 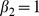, 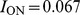, and 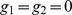. It is apparent that for an optimal noise level (Fig. 6b) the circuit indeed performs the logic AND/NAND function, and that for the smaller (Fig. 6a) and larger (Fig. 6c) noise values faithful logic response is lost. At the low noise (Fig. 6a) the outputs sometimes fail to respond to the 0 to 1 transition from the AND of 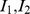, and for the 1 to 0 transition the outputs often wait until *both* inputs go low before responding thereby causing a delayed response. In Fig. 6b the responses are quick for both the up and down transition. Figure 6c shows that at the high noise level the responses are again quick as in 6b, but the outputs also make erroneous transitions. The circuit behavior is seen to be in agreement with the simulations of Eqs. (2)–(3).

**Figure 7:**
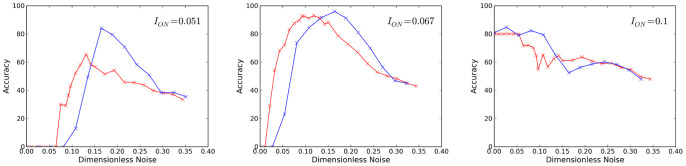
Accuracy 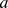 of the AND/NAND logic response for simulations (red) and circuit measurements (blue) as a function of noise strength 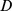 and encoding amplitude 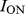. Noise strength 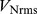 for the circuit measurements has been converted to dimensionless strength 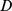 using Eq. (10) with 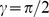.

In order to investigate the range of noise strengths which produce accurate logic response, and to find optimal values for the amplitude 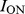 of the small signal inputs 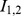 we define an accuracy measure 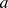,
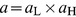
(21)where 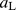 is the percent of time the 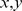 outputs are correct when the AND operation of 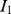 and 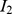 is low, and 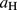 is the percentage of time correct when the AND operation is high. This definition has the property that when the 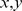 outputs do not respond at all, then 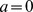 since 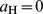 even though 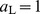. The expectation then is that for no noise there should be no stochastic resonance response so that 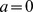, and that for extreme noise each accuracy would approach 50% so that 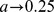. If 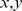 respond immediately with no mistakes then 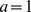. Figure 7 shows accuracy 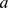 for simulations and circuit measurements as a function of noise strength for different values of encoding amplitude 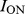.

Figure 7a shows that for a small value of encoding amplitude, 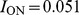 the network is not able to give a faithful logic response at any noise level. The response at low noise and at high noise are as predicted, 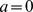 and 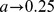, respectively, but the peak of the window of stochastic resonance response is well below 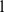. Figure 7b shows a window of noise providing faithful response for 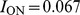. The reason that the accuracy 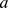 is slightly below 1 in the window is that the 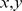 outputs do not respond immediately to the AND/NAND transitions. This time lag causes the percent of time with incorrect response to be non-zero and therefore 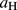 and 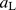 are less than 1. In principle an allowance for a time lag could be included in the calculation of 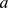 if it were deemed necessary. However, such an added complication would not make the noise window any more apparent than it already is in Fig. 7. Figure 7c shows that at a high value, 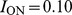, the outputs respond even with no noise, and the addition of noise only creates more errors. The relative shift between the simulation and circuit accuracies is due to assumptions made about the noise spectral density function and the integration in Eq. (16) leading to Eq. (20) which gives the connection between the measured noise amplitude 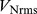 and dimensionless noise 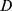. In the idealized case Eq. (16) finds 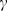 ranges from 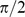 to 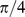 in Eq. (20), with 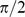 being appropriate for the noise levels used in Fig. 6. Adjusting the value of 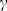 can eliminate the relative shift, but there is nothing to be gained since the appearance of an optimal noise window for LSR at an appropriate value of 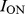 is already apparent.

**Figure 8:**
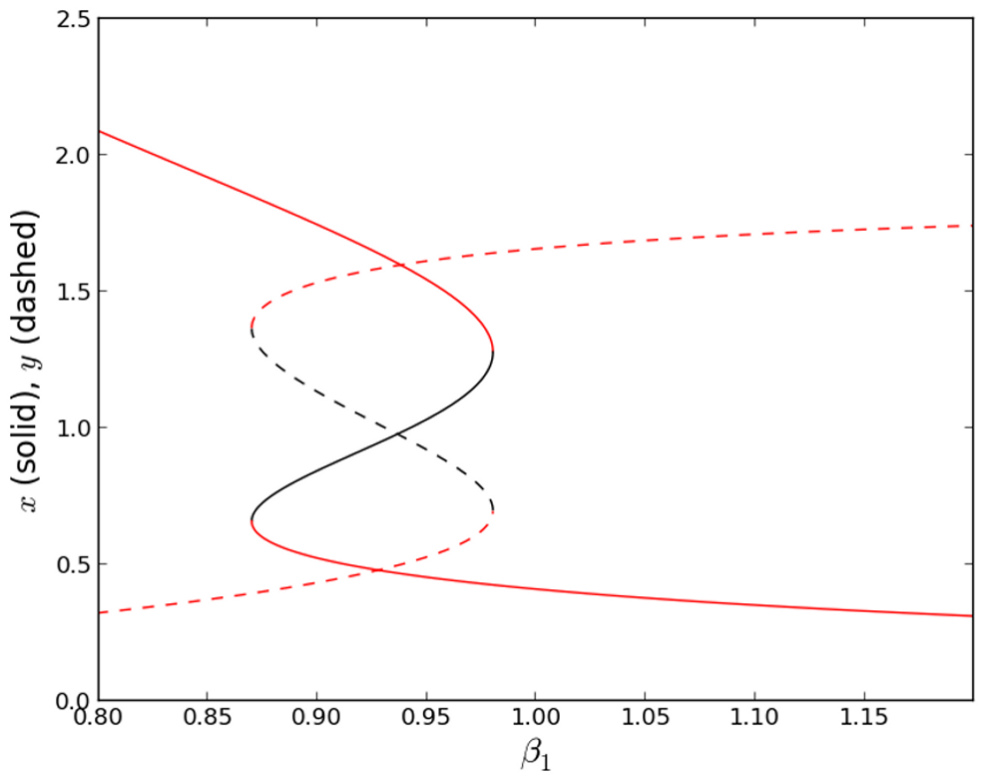
Bifurcation diagram for state-variables 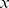 (solid) and 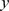 (dashed) configured for OR/NOR. 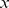
 and 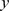 are complementary outputs, when one is high the other is low. Red (black) indicate stable (unstable) fixed points. System is bistable for 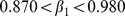. Calculated for Eqs. (2)–(3) with 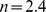, 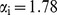, 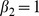, 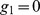, 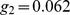, 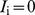, and 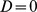.

**Figure 9:**
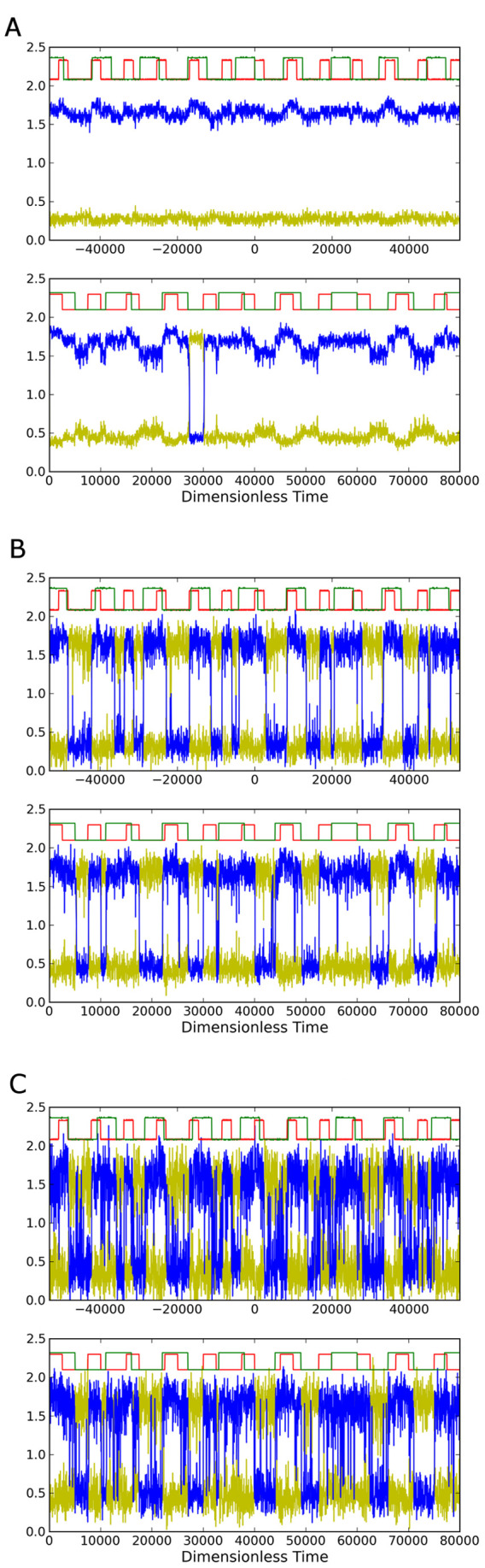
Time series for circuit measurements (upper graph) and simulations (lower) for different noise strengths showing OR/NOR LSR. Panel (b) has noise level within the optimal range for displaying OR/NOR characteristics. Noise strengths in simulation and in circuit are: (a) 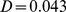 and 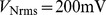, (b) 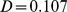 and 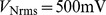, (c) 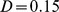 and 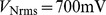. Voltages and times for the circuit measurements have been converted to dimensionless quantities as described in the text.

One can also *reconfigure* the system to another set of logic functions, namely the fundamental OR/NOR logic, by simply including a non-zero value for 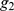. The parameter 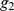 effectively changes the relative position and depth of the wells of the bistable system, allowing the response to morph from AND/NAND to OR/NOR. For instance changing 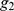 from 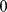 to 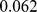 (with all other parameters unchanged) changes the bifurcation diagram from that in Fig. 1 to Fig. 8 showing that the system is now bistable for 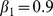. The result is that the system displays a clear OR and the complementary NOR response as shown by the simulation and circuit results in Fig. 9. The low noise case Fig. 9a shows that at this low noise level the system usually fails to respond. Figure 9a also shows the resting states are reversed from the 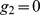 case in Fig. 6a. Figure 9b shows the OR/NOR response at a noise value within the window for LSR, and Fig. 6c shows errors when the noise is too large.

In summary, our results show that the dynamics of the two variables 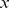 and 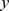 with 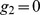, mirror AND and the complementary NAND gate characteristics. Further, when 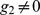, we obtain a clearly defined OR/NOR gate. Since 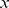 is low when 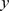 is high and vice-versa, the dynamics of the two variables always yield *complementary* logical outputs, simultaneously. That is, if 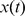 operates as NAND/NOR, 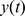 will give AND/OR.

These results extend the scope and indicate the generality of the recently observed phenomena of Logical Stochastic Resonance through experimental verifications. Further, these observations may provide an understanding of the information processing capacity of synthetic genetic networks, with noise aiding logic patterns. It also may have potential applications in the design of biologically inspired gates with added capacity of reconfigurability of logic operations.

We have also demonstrated that the electronic circuit provides an additional tool for investigating dynamics of proposed genetic networks. The circuit measurements are complementary to numerical simulations, thereby giving indication of the robustness of a particular network design and potential for successful realization in a biological system.

Thus the results presented in this work suggest new directions in biomolecular computing, and indicate how robust computation may be occurring at the scale of regulatory and signalling pathways in individual cells. Design and engineering of such biologically inspired computing systems not only present new paradigms of computation, but can also potentially enhance our ability to study and control biological systems [26].
